# *Ginkgo biloba* Extract Improves Insulin Signaling and Attenuates Inflammation in Retroperitoneal Adipose Tissue Depot of Obese Rats

**DOI:** 10.1155/2015/419106

**Published:** 2015-04-16

**Authors:** Bruna Kelly Sousa Hirata, Renata Mancini Banin, Ana Paula Segantine Dornellas, Iracema Senna de Andrade, Juliane Costa Silva Zemdegs, Luciana Chagas Caperuto, Lila Missae Oyama, Eliane Beraldi Ribeiro, Monica Marques Telles

**Affiliations:** ^1^Departamento de Ciências Biológicas, Universidade Federal de São Paulo (UNIFESP), Rua Arthur Riedel, 275 Eldorado, 09972-270 Diadema, SP, Brazil; ^2^Departamento de Fisiologia, Disciplina de Fisiologia da Nutrição, Universidade Federal de São Paulo (UNIFESP), Rua Botucatu, 862 Vila Clementino, São Paulo, SP, Brazil

## Abstract

Due to the high incidence and severity of obesity and its related disorders, it is highly desirable to develop new strategies to treat or even to prevent its development. We have previously described that *Ginkgo biloba* extract (GbE) improved insulin resistance and reduced body weight gain of obese rats. In the present study we aimed to evaluate the effect of GbE on both inflammatory cascade and insulin signaling in retroperitoneal fat depot of diet-induced obese rats. Rats were fed with high fat diet for 2 months and thereafter treated for 14 days with 500 mg/kg of GbE. Rats were then euthanized and samples from retroperitoneal fat depot were used for western blotting, RT-PCR, and ELISA experiments. The GbE treatment promoted a significant reduction on both food/energy intake and body weight gain in comparison to the nontreated obese rats. In addition, a significant increase of both Adipo R1 and IL-10 gene expressions and IR and Akt phosphorylation was also observed, while NF-*κ*B p65 phosphorylation and TNF-*α* levels were significantly reduced. Our data suggest that GbE might have potential as a therapy to treat obesity-related metabolic diseases, with special interest to treat obese subjects resistant to adhere to a nutritional education program.

## 1. Introduction

The incidence of both obesity and overweight has been dramatically increasing around the world. In 2008, 35% of worldwide population was overweight while 11% was obese [1]. In addition, it has been estimated that obesity will achieve one-third of the population in 2030 [2]. This perspective is particularly worrying since obesity is related to chronic comorbidities, such as insulin resistance, type 2 diabetes (T2D), subclinical inflammation, and others [3].

It has been suggested that consumption of high-fat diet is directly involved in the obesity pathogenesis since it affects either central control of food intake and peripheral metabolism, resulting in increased body weight gain, insulin resistance, and other metabolic disturbances [4, 5]. Thus, we have previously demonstrated that prolonged hyperlipidic diet ingestion promoted in rats a significant increase of body adiposity, triacylglycerol, and glucose plasma levels with a concomitant loss of insulin sensitivity [6].

Insulin resistance is a chronic condition in which the hormone insulin fails to activate its own signaling cascade, resulting in hyperglycemia. It has been highly correlated to visceral adiposity excess and increased white adipose tissue (WAT) expression of cytokines, such as tumor necrosis factor-alpha (TNF-*α*) and interleukin-6 (IL-6) [3, 7]. Furthermore, high-fat diet intake has been pointed as an important risk factor for insulin resistance, since it both impairs insulin signaling pathway and stimulates inflammation, via Toll-like receptors signaling cascade [5, 7, 8].

Taking into account that most of hypoglycemiants present undesirable side effects [9–12] and due to the severity of insulin resistance progression it is highly desirable to discover new drugs and treatment methods. It has been proposed that* Ginkgo biloba *extract (GbE) might have positive effects on hyperglycemia. This plant extract mainly contains around 24% flavonoid glycosides and 6% terpenoids, including A, B, C, M, J, P, and Q ginkgolides [13].

We have previously described that prolonged GbE treatment significantly reduced food intake and body adiposity, prevented against hyperglycemia and dyslipidemia, while it increased insulin sensitivity evaluated by ITT (insulin tolerance test) in obese rats fed with lard-enriched hyperlipidic diet [6]. In agreement to our previous findings, other studies proposed that GbE intake improved glycaemic profile of both healthy and T2D patients [14, 15]. In addition, a reduction on glucose elevation stimulated by oral administration of saccharin agents in rats was demonstrated [16].

The data above suggest beneficial effects of GbE on insulin resistance and obesity-related disorders. However, it is highly important to better describe the mechanisms by which GbE improves insulin action. In this context, the present study aimed to evaluate if a 14-day oral GbE treatment alters retroperitoneal WAT depot insulin and Toll-like receptors signaling cascades of diet-induced obese rats, a model of insulin resistance.

## 2. Materials and Methods 

### 2.1. Animals

The Committee on Animal Research Ethics of the Universidade Federal de São Paulo approved all procedures for the care of the animals used in this study (Process number: 271359). All efforts were made to minimize suffering. Male Wistar rats from CEDEME (São Paulo, Brazil) were housed 4 per cage and maintained in controlled conditions of light (12 : 12-h light/dark, lights on at 6 am) and temperature (23°C ± 1°C), with free access to food and water.

2-month-old rats were fed a highly fat-enriched diet which was prepared by adding 40% (w/w) standard chow plus 28% (w/w) lard, 2% (w/w) soy oil, 10% (w/w) sucrose, 20% (w/w) casein, in order to obtain the protein content of the control diet, and butylated hydroxytoluene in the amount of 0.02% (w/w) of the additional oil. This provided 19.5% of energy as carbohydrate, 23.2% as protein, and 57.3% as fat.  Table 1 shows the macronutrient and fatty acid compositions of the diet.

After 8 weeks, animals were divided into two groups, according to the phytotherapy treatment described below.

### 2.2. Phytotherapy Treatment

*Ginkgo biloba* extract (GbE) was obtained from Southern Anhui Dapeng (China) and contained 26.12% of flavone glycosides, 6.86% of terpenoids, 2.20% of ginkgolide A, 1.11% of ginkgolide B, 1.05% of ginkgolide C, and 2.50% of bilobalide.

Phytotherapy treatment was performed for a 14-day period. The obese animals were divided in two groups: O+V (Obese + Vehicle) and O+Gb (Obese +* Ginkgo biloba*). The O+Gb group was daily gavaged with 500 mg/kg of GbE [17, 18] diluted in 1 mL of 0.9% saline (vehicle) while the O+V was gavaged with 1 mL of vehicle.

### 2.3. Body Weight Gain, Accumulated Food, and Energy Intake

During the phytotherapy treatment period, 24-h food intake and body weight were daily measured. The evaluation of food intake was calculated by the difference between the amount of meal offered and the remnant after 24 hours.

Body weight gain was calculated by the difference between final weight (last day of treatment) and initial weight (first day of treatment). Accumulated food and energy intakes were measured by the mean of the first 13 days of treatment. In the last day of treatment rats were kept overnight fasted.

### 2.4. Retroperitoneal Adipose Tissue and Serum Parameters

Rats were anesthetized with sodium thiopental (80 mg/Kg of body wt, intraperitoneal) and decapitated after a 10-hour fasting period. Retroperitoneal white adipose tissue depot was removed and homogenized in 1.0 mL of lysis buffer (100 mM Tris, pH 7.5, 10 mM EDTA, and 0,1 mg/mL aprotinin; 2 mM PMSF; 10 mM sodium orthovanadate; 100 mM sodium fluoride; 10 mM sodium pyrophosphate; and 10% TritonX-100). Levels of proinflammatory cytokine TNF-*α* and anti-inflammatory cytokine IL-10 were measured by ELISA kit (R&D Systems).

Portal vein blood was also collected for the measurement of adiponectin by Milliplex MAP (Millipore).

### 2.5. Western Blotting

Rats were deeply anesthetized with sodium thiopental (80 mg/Kg of body wt, intraperitoneal). The abdominal cavity was opened and negative control samples (O+V− and O+Gb−) were obtained from the left side retroperitoneal fat depot. After the collection, samples were immediately inserted into a vial containing 3.0 mL of lysis buffer (100 mM Tris, pH 7.5, 10 mM EDTA, and 0.1 mg/mL aprotinin; 2 mM PMSF; 10 mM sodium orthovanadate; 100 mM sodium fluoride; 10 mM sodium pyrophosphate; and 10% TritonX-100), homogenized, and centrifuged at 16000 g for 40 minutes at 4°C. Then, the portal vein was exposed and 10^−5^ M of insulin was injected intravenously (i.v.). Right side retroperitoneal fat depot was removed 90 seconds after the i.v. insulin injection (positive samples: O+V+ and O+Gb+) following the same protocol described above [19, 20]. Total protein was quantified by BCA kit (BioRad) and samples were used for both immunoprecipitation and total extract evaluations.

To reduce the risk of nonspecific antibody binding, we evaluated the IR phosphorylation levels after immunoprecipitation with antibody against IR. To perform immunoprecipitation experiments, samples were overnight incubated with 10 *µ*L primary antibody anti-IR (insulin R*β* sc-711) and proteins were precipitated by Protein A Sepharose (GE). After all, proteins were separated on 10% SDS-PAGE. Proteins were then transferred to nitrocellulose membranes by wet transfer apparatus (Bio-Rad). The membranes were preincubated for 1 hour in blocking buffer (5% bovine serum albumin [BSA], 1 M Tris, pH 7.5, 5 M NaCl, and 0.02% Tween-20). Membranes were overnight incubated at 4°C with the primary antibody against p-Tyr (Cell Signaling 8954). All membranes were then incubated with specific horseradish peroxidase-conjugated anti-rabbit IgG antibody (Cell Signaling 7074) followed by chemiluminescence detection (Amersham Biosciences). Since all samples were immunoprecipitated with IR antibody, we considered that bands with molecular weight of 95 kDa were related to the phosphorylated form of IR. In addition, IR levels were used as internal standards since all the other proteins were removed by the immunoprecipitation method.

To perform the total extract experiments, after the protein quantification, total proteins were then separated on 8% SDS-PAGE. Proteins were transferred by semidry transfer apparatus (Bio-Rad).

All membranes were overnight incubated at 4°C with the primary antibody against phospho-Akt (Cell Signaling Ser 473–9271); Akt (Cell Signaling 9272), phospho-NF-*κ*B p65 (Cell Signaling Ser 536–3033), NF-*κ*B p65 (Cell Signaling 6956), MyD88 (Cell Signaling 4283), TLR4 (SC 293072), and *β*-tubulin (Cell Signaling 2146). All membranes were then incubated with specific horseradish peroxidase-conjugated anti mouse/rabbit IgG antibody (Cell Signaling 7076; Cell Signaling 7074, resp.) followed by chemiluminescence detection (Amersham Biosciences). *β*-tubulin (Cell Signaling 2146) level was used as an internal standard.

Quantitative analysis was performed with Scion Image software (Scion Corporation, Frederick, MD, USA). In all experiments, at least one sample from each group was analyzed simultaneously and the results were expressed as percentage change relative to the basal levels.

### 2.6. RNA Extraction and Quantitative Real-Time Polymerase Chain Reaction (qPCR)

In order to evaluate the gene expression of Adipo R1, Adipo R2, and IL-10, additional groups (O+V and O+Gb) of five rats each were performed. For total RNA extraction, two hundred mg of frozen retroperitoneal adipose tissue from each sample were homogenized by adding 1 mL of Trizol reagent (Invitrogen, USA). The samples were centrifuged at 16.000 g for 15 min at 4°C and the aqueous phase was removed and mixed with 0.5 mL of isopropyl alcohol. After centrifugation at 16.000 g for 10 min at 4°C, the pellet was washed with 1 mL of 75% ethanol and then dissolved in 20 *µ*L DEPC-Treated water (Ambion, USA).

One microgram of RNA was reverse transcribed to cDNA using the High-Capacity cDNA kit (Applied Biosystems). Gene expression was evaluated by real-time qPCR using the Taqman PCR Assays. Primers and probe catalog numbers were Adipo R1 (Rn01483784_m1), Adipo R2 (Rn01463173_m1), IL-10 (Rn00563409_m1), and Actin b (Rn00667869_m1).

Reactions were performed in 96-well plates and carried out in triplicate. Amplification conditions consisted of 40 cycles of 50°C/2 min, 95°C/10 min, 95°C/15 s, and 60°C/1 min. The method 2^−ΔΔCt^ was used to evaluate the relative quantification of amplification products.

### 2.7. Statistics

Statistical analysis was performed using PASW Statistics version 19 (SPSS Inc, Chicago, IL, USA) with the level of statistical significance set at *P* < 0.05. Comparisons among two groups were performed by Student's *t* test.

## 3. Results

### 3.1. Food Intake and Body Adiposity in Response to Phytotherapy Treatment

**Figure 1:**
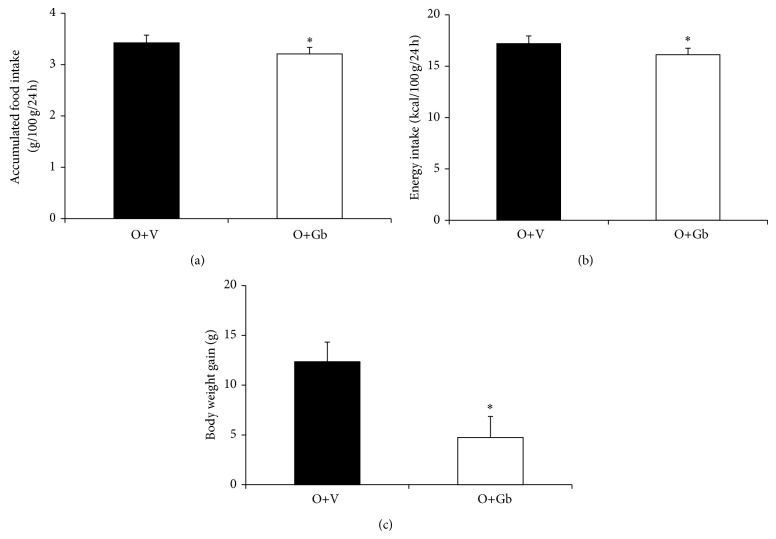
Food intake and body weight gain in response to EGb treatment. (a) Accumulated food intake (g/100 g/24 h), (b) energy intake (Kcal/100 g/24 h), and (c) body weight gain (g) of O+V (*n* = 17) and O+Gb (*n* = 15) groups during the phytotherapy treatment. ^*^ *P* < 0.05 versus O+V.

Accumulated food intake during the first 13 days of phytotherapy treatment is illustrated in Figure 1(a). It is interesting to note that O+Gb group ingested 6.3% less than O+V group (*P* = 0.031). In relation to energy intake, it can be observed at Figure 1(b) that O+Gb also presented a significant reduction of 6.3% in comparison to O+V (*P* = 0.031).

The effect of GbE on body weight gain is presented in Figure 1(c). It can be seen that the O+Gb group had a significant reduction of 62% (*P* = 0.013) in comparison to O+V group.

### 3.2. Cytokine Levels and Gene Expression

Table 2 presents the results of retroperitoneal fat depot cytokine levels. A decrease of 36% (*P* = 0.014) on TNF-*α* was observed in the O+Gb in comparison to the O+V group. The levels of IL-10 and IL-6 were similar in both groups.

**Figure 2:**
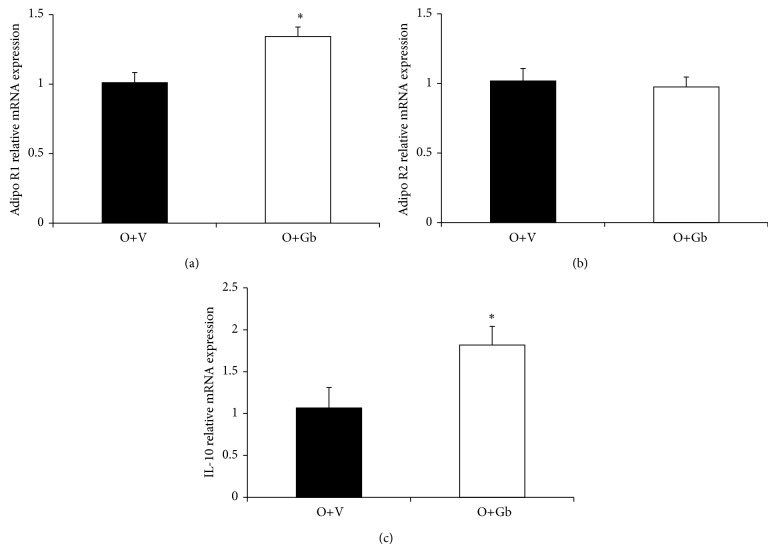
Effect of GbE on retroperitoneal fat depot gene expression of Adipo R1, Adipo R2, and IL-10. Gene expression in retroperitoneal WAT depot of O+V (*n* = 5) and O+Gb (*n* = 5) groups evaluated by Real Time PCR. ^*^ *P* < 0.05 versus O+V.

Figure 2 depicts the effect of GbE on retroperitoneal fat depot gene expression of Adipo R1, Adipo R2, and IL-10. It can be observed in Figures 2(a) and 2(c) that the GbE treatment promoted a significant increase on gene expression of both Adipo R1 (33%; *P* = 0.013) and IL-10 (70%; *P* = 0.040), in comparison to the O+V group. However, no differences were observed in gene expression of Adipo R2 in response to GbE treatment (Figure 2(b)).

### 3.3. Fasting Serum Adiponectin Levels

In relation to adiponectin serum levels, no differences were observed among O+V group (14.74 ± 0.92 *µ*g/mL) and O+Gb group (12.96 ± 1.16 *µ*g/mL).

### 3.4. IR and AKT Phosphorylation Levels

**Figure 3:**
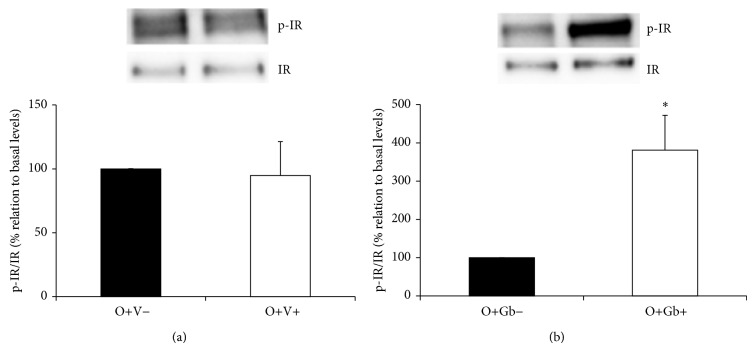
Effect of GbE on IR phosphorylation levels of retroperitoneal fat depot: insulin-induced IR phosphorylation levels in retroperitoneal WAT depot of groups: (a) O+V− (*n* = 10) and O+V+ (*n* = 9); (b) O+Gb− (*n* = 9) and O+Gb+ (*n* = 9) evaluated by western blotting. ^*^ *P* < 0.05 versus basal levels.

In Figure 3(a) it can be observed that insulin-induced IR phosphorylation (O+V+) was impaired by the ingestion of high-fat diet, since no differences were observed in relation to basal levels (O+V−). However, it can be seen in Figure 3(b) that prolonged administration of GbE promoted a significant 2.81-fold increase (*P* = 0.004) on insulin-induced IR phosphorylation (O+Gb+) in relation to basal levels (O+Gb−).

**Figure 4:**
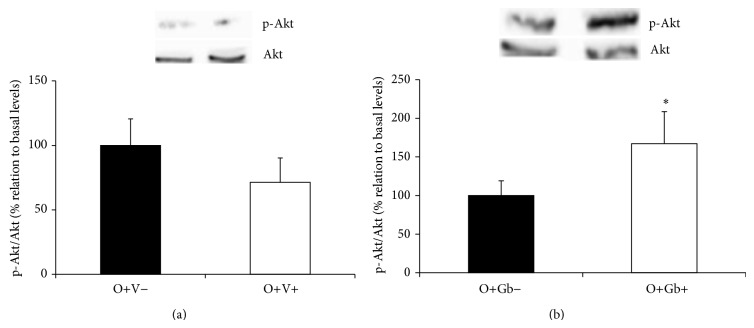
Effect of GbE on Akt phosphorylation levels of retroperitoneal fat depot: insulin-induced Akt phosphorylation levels in retroperitoneal WAT depot of groups: (a) O+V− (*n* = 8) and O+V+ (*n* = 9); (b) O+Gb− (*n* = 8) and O+Gb+ (*n* = 7) evaluated by western blotting. ^*^ *P* < 0.05 versus basal levels.

Figure 4 illustrates that Akt phosphorylation was also stimulated by the GbE treatment. The GbE treatment promoted a significant 0.67-fold increase (*P* = 0.039) on Akt phosphorylation levels in comparison to basal levels (O+Gb+ versus O+Gb−) (Figure 4(b)) whilst no effect was observed in nontreated obese rats after insulin infusion (O+V+ versus O+V−) (Figure 4(a)).

### 3.5. Inflammatory Signaling Pathway

**Figure 5:**
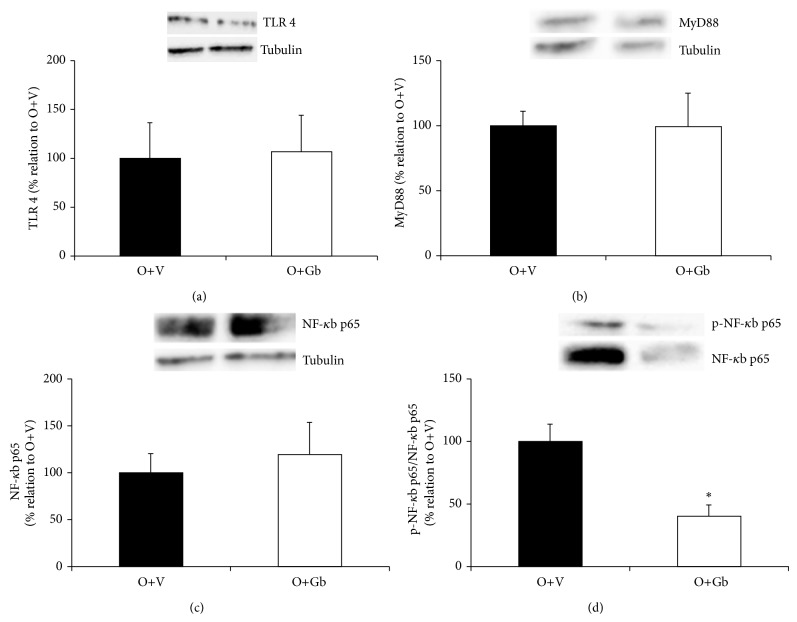
Effect of GbE on inflammatory signaling pathway: total protein levels of TLR4 (O+V *n* = 6; O+Gb *n* = 6), MyD88 (O+V *n* = 13; O+Gb *n* = 9), NF-*κ*B p65 (O+V *n* = 5; O+Gb *n* = 4), and phosphorylation of NF-*κ*B p65 (O+V *n* = 10; O+Gb *n* = 8) in retroperitoneal WAT depot evaluated by western blotting. ^*^ *P* < 0.05 versus O+V.

It can be seen in Figure 5 that GbE treatment did not modify the total protein levels of TLR4, MyD88, and NF-*κ*B p65 (*P* = 0.900; *P* = 0.982; *P* = 0.163, resp.) in retroperitoneal fat depot. Yet, the GbE treatment did significantly reduce the phosphorylation of NF-*κ*B p65 by 60% in comparison to the nontreated obese rats (*P* = 0.004).

## 4. Discussion

It has been considered that prolonged fat intake is the main predisposing risk factor for the development of obesity [21, 22]. High fat intake also impairs insulin action by reducing glucose uptake and both IR and Akt phosphorylation in brown and white adipose tissues [5, 6, 23]. Due to the risks involved in the obesity and insulin resistance establishment, it is highly desirable to develop new strategies to treat obesity and its related disorders.

In our previous study it was demonstrated that prolonged treatment with GbE promoted a significant visceral adiposity loss, improvement of insulin sensitivity, reduction of dyslipidemia, and stimulation of insulin signaling cascade in gastrocnemius muscle [6]. Taking into consideration the promising results obtained in our previous study, the present one was aimed to further evaluate the beneficial effects of GbE on obesity-related insulin resistance, focusing now on both insulin and inflammatory cascades of retroperitoneal fat depot, an insulin-dependent tissue.

Similar to our previous study [6], the present data has demonstrated that GbE treatment significantly has decreased food/energy intake and, in addition, it has also reduced the body weight gain of diet-induced obese rats. Data on literature are scarce to demonstrate such effect. However, some studies demonstrated a potent anti-inflammatory effect of GbE (24–26) especially via reduction of LPS-induced inflammatory cytokines or inhibition of the Toll-like receptors pathway (27–30). Since obesity is related to hypothalamic inflammation (31–35), it is possible that the treatment with GbE might have promoted a positive anti-inflammatory effect on hypothalamus, increasing anorexigenic peptides levels and/or reducing the orexigenic ones resulting in appetite suppression and weight loss. Additional studies are necessary to better comprehend the mechanisms involved in the GbE-induced appetite suppression of obese rats.

In the nontreated obese group insulin failed to stimulate the phosphorylation of both IR and Akt in retroperitoneal fat depot indicating that high fat intake impairs insulin signaling. Interestingly, in the obese group treated with GbE, the phosphorylation of both IR and Akt was significantly increased by 281% and 67%, respectively. It is noteworthy that the beneficial effects of GbE were observed in rats that remained fed with high fat diet, suggesting that it might be efficient to treat the development of obesity-related insulin resistance.

Previous study of our laboratory showed that GbE improved insulin sensitivity evaluated by the insulin tolerance test while it did significantly improve insulin-induced Akt phosphorylation and IRS-1 levels with a concomitant reduction on PTP-1B levels in gastrocnemius muscle [6]. In addition, other studies have shown that GbE reduces glycaemia and improves glucose intolerance [16, 24]. Besides, GbE stimulated both pancreatic beta-cells function and insulin production in healthy subjects with normal glucose tolerance, while it significantly reduced the glycated hemoglobin levels of T2D patients after a 3-month period of treatment [14, 15].

It is well described that adiponectin—an adipokyne expressed inversely to body adiposity—improves insulin signaling and reduces inflammation especially via Adipo R1 receptor [25, 26]. We failed to demonstrate an effect of GbE on the adiponectin serum levels. However, the present study has demonstrated a significant increase on the adiponectin receptor, Adipo R1, gene expression in retroperitoneal fat depot while no effect was observed on the Adipo R2, indicating that GbE might improve the signaling of adiponectin. In agreement with our data, Liu et al. [27] revealed that the GbE fraction isoginkgetin enhances adiponectin secretion* in vitro*, suggesting a positive effect of GbE on the adiponectin antidiabetic action. In addition, Rasmussen et al. [25] described that the weight loss observed in obese subjects submitted to a hypocaloric diet was associated with an increase in Adipo R1 mRNA levels. Yamaguchi et al. [26] demonstrated that the binding of adiponectin to the Adipo R1 receptor, but not to Adipo R2, in macrophages was responsible for the inhibition of TLR signaling pathway mediated by the suppression of NF-*κ*B. In view of the above considerations, it is possible that the increased expression of Adipo R1 herein demonstrated might have contributed for the stimulatory effect of GbE on insulin signaling.

Another important factor involved in the pathogenesis of insulin resistance is the low grade inflammation present in obese subjects [28]. It has been shown that, in this condition, the proinflammatory adipokine TNF-*α* is increased while a reduction can be observed in the levels of the anti-inflammatory IL-10, resulting in the impairment of insulin sensitivity and glucose uptake [29].

Despite the fact that, in the present study GbE failed to alter TLR4, MyD88, and NF-*κ*B p65 proteins expression, it has significantly reduced the phosphorylation of NF-*κ*B p65 in retroperitoneal fat depot, indicating an inhibitory effect on this inflammatory pathway. In fact, Yoshikawa et al. [30] described GbE as a potent anti-inflammatory agent. The majority of GbE anti-inflammatory effects were observed by LPS induction while the effect of GbE on the obesity-related inflammation has remained unclear. Thus, the present study is the first to demonstrate a beneficial role of GbE in such condition.

The present data have also shown that GbE reduced TNF-*α* levels while IL-10 and IL-6 levels were not modified in retroperitoneal adipose tissue. Besides, our results have also demonstrated an increase on the anti-inflammatory cytokine IL-10 gene expression in retroperitoneal fat depot. It is possible that the GbE treatment duration was not sufficient to affect the other cytokine levels rather than TNF-*α*. In addition, it is well known that the white adipose tissue presents a depot-specific response to different stimuli [31, 32]. It allows to speculate that other fat depots rather than the retroperitoneal one might present altered levels of IL-6 and IL-10 in response to GbE treatment.

It is known that increased plasma IL-10 levels are associated with visceral reduction [33]. Furthermore, IL-10 improves insulin sensitivity and glucose transport, thereby having a protective role against obesity-induced insulin resistance [29, 34]. In addition, the low IL-10 production capacity presented in pathological conditions such as obesity is associated with the development of metabolic syndrome and T2D [35]. In this context, it is possible that, in a more prolonged treatment period, the stimulatory effect of GbE on IL-10 gene expression herein demonstrated might also lead to an increase on IL-10 tissue levels, contributing to the beneficial effects of GbE on insulin signaling cascade already observed after 14 days of treatment.

An inhibitory effect of GbE on TNF-*α* levels on other tissues, such as brain and lungs, has been described [36, 37]. We consider that the anti-inflammatory effect of GbE via reduction of TNF-*α* retroperitoneal fat depot levels might soften the harmful effects of the prolonged consumption of high fat diets, resulting in the stimulation of the insulin signaling pathway.

## 5. Conclusions

The data presented above showed that GbE markedly stimulated the insulin signaling cascade, since it promoted the insulin-induced phosphorylation of both IR and Akt in retroperitoneal fat depot. Nevertheless, our results indicate that the inhibitory effect of GbE on both NF-*κ*B p65 phosphorylation and TNF-*α* levels might have contributed to the stimulation of the insulin signaling. Summing up, the results herein presented suggest a potential use of GbE to treat obesity-related insulin resistance. These results are especially interesting taking into consideration the high number of obese people resistant to perform diet therapy. However, additional studies are necessary to better comprehend the effects of GbE on obesity-related disorders.

## Figures and Tables

**Table 1:** Macronutrients and fatty acid compositions of high fat diet.

	High fat diet

Humidity (%)	1.1
Lipid (%)	31.6
Protein (%)	27.0
Carbohydrate (%)	27.5
Total food fiber (%)	8.6
Mineral residue fixed (%)	4.2
Sodium chloride (%)	0.2
Calculated energy (Kcal/g)	5.0

**Table 2:** Retroperitoneal fat depot cytokine levels (*ρ*g/*µ*g of protein).

Cytokine	O + V	O + Gb
IL-10	0.47 ± 0.09	0.33 ± 0.03
IL-6	0.57 ± 0.09	0.55 ± 0.08
TNF-*α*	0.47 ± 0.07	0.30 ± 0.02^*^

^*^ *P* < 0.05 versus O + V.
